# Editorial: Bioactive compounds in mineral bioavailability: Activities, structures, and mechanisms

**DOI:** 10.3389/fnut.2022.1050670

**Published:** 2022-10-18

**Authors:** Tao Hou

**Affiliations:** College of Food Science and Technology, Huazhong Agricultural University, Wuhan, China

**Keywords:** minerals, absorption, bioactive compounds, structures, mechanisms

Minerals such as calcium, zinc, iron, and copper are extremely crucial for human health and possess a wide variety of biological functions (Figure 1). For instance, calcium is the most abundant inorganic element in human body accounting for 1.5–2.2% of total body weight. In addition, calcium is the relevant component participates in cell metabolism, bone growth, blood coagulation, nerve conduction, muscle contraction and cardiac function. Calcium is also responsible for many important physiological functions such as cell proliferation, responses to hormones and the release of neurotransmitters. Iron is mainly involved in generation and oxygen transportation of hemoglobin. Meanwhile, iron is a vital substrate for hemoglobin production, and sufficient iron stores are necessary to achieve and maintain adequate levels of hemoglobin. Zinc is a catalytic component of a large number of enzymes and has a structural and biological role in many proteins, peptides, hormones, transcriptional, and growth factors and cytokines. Copper, as an essential element, plays a vital role as a cofactor for a variety of enzymes. However, copper is capable of producing reactive oxygen species inducing DNA strand breaks and oxidation of nucleotide bases. In a word, minerals play important roles in human health.

**Figure 1:**
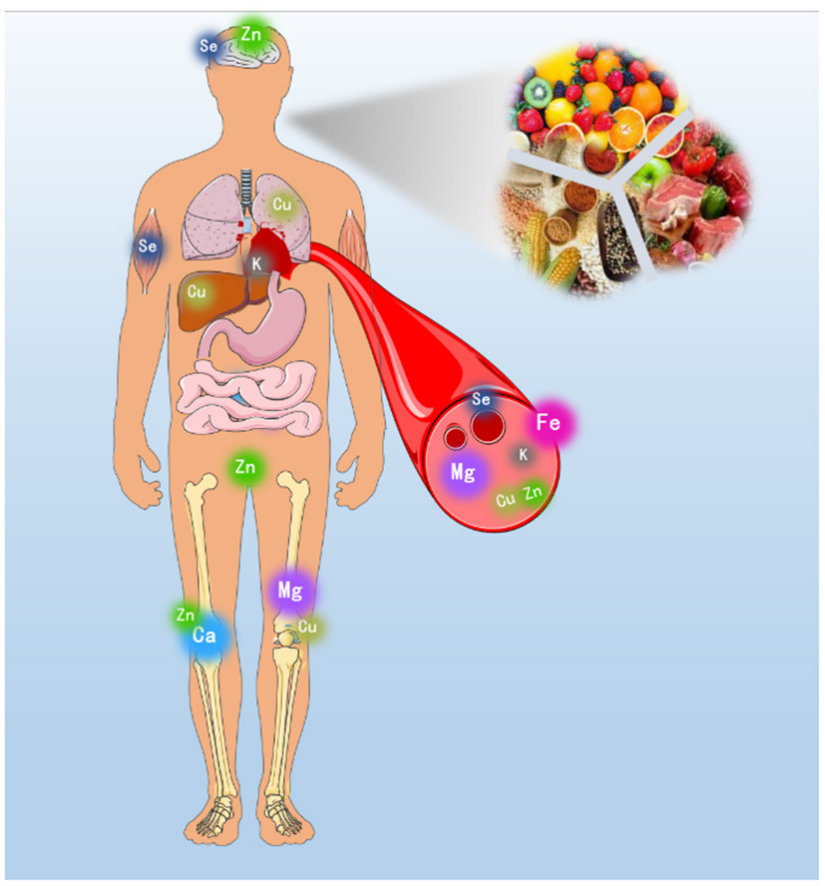
Minerals possess a wide variety of biological functions for human health.

Deficiencies of dietary minerals can lead to many diseases. Insufficient calcium intake can result in a release of calcium from bone and increases the risk of osteoporosis. Iron deficiency in human body can consequently lead to iron deficiency anemia and neurological disorders. Zinc deficiencies give rise to alopecia, diarrhea, delayed sexual maturation as well as eczematous skin rash. Therefore, a majority of calcium and ferrous ions nutritional fortifiers have been developed to improve mineral deficiency.

However, many factors, such as dietary structures, environments, processing methods of food, and habits, can drive people to suffer from mineral deficiency. For instance, many staple foods in the diet, such as cereals, corn, rice and legumes, often contain phytate. Phytate can lead to mineral deficiencies because it acts as an inhibitor of zinc, calcium and iron absorption. In addition, the low bioavailability of mineral is a vital factor which can limit the absorption of minerals. Metal ions may interact with each other, which in turn may result in a decrease in their bioavailability. Dietary calcium may significantly diminish the absorption of ferrous and ferric iron in a dose-related manner. Individuals consuming a high calcium diet containing marginal amounts of iron can develop an iron deficiency. The bioavailability of zinc can be markedly affected or reduced by ingestion of large amounts of other elements such as iron and copper. Iron can interfere with the absorption of calcium, magnesium and zinc if, for example, iron and multivitamins are taken at the same time.

Amino acid chelators, such as ferrous bisglycinate, have been developed commercially and reported to protect iron from dietary inhibitors and to have a fourfold higher iron absorption than ferrous sulfate. Amino acids, such as histidine and methionine, have been used in an effort to enhance zinc bioavailability due to their ability to increase zinc solubility. However, these amino acids were gradually replaced due to their poor transport capacity in the intestine, tendency to cause unwanted color reactions and to provoke fat oxidation. Therefore, dietary intake of bioactive compounds to improve the bioavailability of minerals is an effective and safe nutritional approach that shows promise to address dietary mineral deficiencies. Studies in this area have risen significantly in recent years. Proteins/peptides, phytochemicals, vitamins, polysaccharides, prebiotics and gut-mineral axis were shown the ability to promote mineral bioavailability and prevent relevant diseases. This Research Topic encourages articles that explore or prepare novel bioactive components associated with minerals in natural products, structure-activity relationships, intestinal behaviors, relevant disease prevention mechanisms, and dietary patterns are also the focuses in this topic.

In this topic we published 4 original Research Topics, which were related to the following subtopics:

- Preparation, characterization, and evaluation of bioactive components with mineral bioavailability promotion activity from natural products.
- The influence of processing methods on the structure, morphology, and minerals release kinetics of food.
- The potential mechanisms for the treatment of related diseases caused by mineral deficiency by bioactive substances and their metabolites.
- Novel efficient and safe mineral delivery systems prepared by natural products.
- The influence of gut-minerals axis on the bioactive functions *in vivo*.

Specifically, Huang et al. reported the preparation and characterization of casein phosphopeptide-calcium chelate (CPP-Ca), and explored the osteogenic activity mechanism through MC3T3-E1 cell model. The results showed that the calcium chelation rate of CPPs was 23.37%, and the calcium content of CPP-Ca reached 2.64 × 105 mg/kg. Compared with the control group, the proliferation of MC3T3-E1 cells treated with 250 μg/mL of CPP-Ca increased by 21.65, 26.43, and 28.43% at 24, 48, and 72 h, respectively, and the alkaline phosphatase (ALP) activity and mineralized calcium nodules of MC3T3-E1 cells were notably increased by 55 and 72%. KEGG pathway indicated that the AMPK, PI3K-Akt, MAPK, and Wnt signaling pathways were involved in the differentiation of MC3T3-E1 cells. This study provided a theoretical basis for CPP-Ca as a nutritional additive in the treatment and prevention of osteoporosis. Wang et al.'s work aimed at assessing the influence of comminuting methods, including colloid mill, planetary ball mill and dynamic high-pressure micro-fluidization on the chemical composition, particle properties, morphology and calcium release of chicken bone. The results showed that, compared with the other groups, samples treated by dynamic high-pressure micro-fluidization released more calcium ions, which was related to the significant effects on surface calcium composition and reducing particle size. Therefore, dynamic high-pressure micro-fluidization has a great potential in the processing of bone-derived products. Wang et al. and Liu et al. studied the different calcium sources or chicken skin-derived collagen peptides-zinc chelate of bioactive functions such as immune performance, diarrhea rate, intestinal barrier, gut microbial, represses tumor growth and invasion *in vivo*, respectively. We hope that this special issue can build a communication platform for global scholars on mineral bioavailability studies, so that cooperative research can be carried out in the future to benefit people all over the world.

## Author contributions

The author confirms being the sole contributor of this work and has approved it for publication.

## Conflict of interest

The author declares that the research was conducted in the absence of any commercial or financial relationships that could be construed as a potential conflict of interest.

## Publisher's note

All claims expressed in this article are solely those of the authors and do not necessarily represent those of their affiliated organizations, or those of the publisher, the editors and the reviewers. Any product that may be evaluated in this article, or claim that may be made by its manufacturer, is not guaranteed or endorsed by the publisher.

